# A qualitative exploration of how a community engagement approach influences community and health worker perceptions related to family planning service delivery in Togo

**DOI:** 10.3389/frph.2024.1389716

**Published:** 2024-07-03

**Authors:** Leanne Dougherty, Sethson Kassegne, Robert Nagbe, Joseph Babogou, Paula Peace, Farida Moussa, Karen Kirk, Hilaire Tokplo, Djibril Ouro-Gnao, Serge Prince Agbodjan, Dana Loll, Timothy R. Werwie, Martha Silva

**Affiliations:** ^1^ Breakthrough RESEARCH, Population Council, Washington, DC, United States; ^2^ CERA Group, Lomé, Togo; ^3^ Population Council, Lomé, Togo; ^4^ JHU CCP, Lomé, Togo; ^5^ Tulane University, Lomé, Togo

**Keywords:** family planning, community engagement, Togo, behavior change, health workers

## Abstract

**Background:**

There is a growing body of evidence that asserts community engagement approaches can improve the quality of reproductive health services. Family planning (FP) programs in Togo are implementing such approaches, which aim to mobilize both health workers and communities to improve FP service quality and FP uptake. However, there is not enough known about the enabling factors and challenges associated with implementation, or the extent to which the programs improve outcomes leading to contraceptive uptake.

**Methods:**

We qualitatively explored how a community engagement approach influenced health worker and community perceptions related to FP service delivery in and around the city of Lomé, Togo, within the context of the broader integration of social and behavior change and service delivery. We conducted 18 in-depth interviews with health workers and 9 focus group discussions with community members.

**Results:**

We found the approach, which included community dialogues, site walkthrough visits and the development of community action plans, worked synergistically together to support collaborative action between communities and health workers to increase mutual understanding of their collective needs related to FP services. Community members cited improved reception at the health facilities by health workers and indicated that the site walkthrough visits created a greater sense of empathy towards the providers and the challenges faced in their work environment. Health workers acknowledged a greater understanding of barriers at the community level following community dialogues, particularly among community members that are not routinely encountered at the health facility for reproductive health services such as men and youth. We found limited implementation of health facility improvements included in community action plans because they were dependent on commitment from community leadership and the need to mobilize additional support or financial resources.

**Conclusion:**

Community engagement approaches are a promising mechanism to support collaboration and enhance mutual understanding between health workers and communities to achieve improved FP service quality. Future programs should consider incorporating additional mechanisms to monitor community action plans and provide support to address structural challenges at the facility level particularly those that require financial resources.

## Background

In Togo, unmet need for contraception is high at 34% (1), and only 23% of all women of reproductive age are currently using a modern method of contraception (2). Previous studies have found that socio-cultural norms, including the belief that male partners make decisions related to women's reproductive health needs (3), as well as a belief among men that family planning (FP) could encourage infidelity and promiscuity among women, are negatively associated with contraceptive use (4). Poor provider training and barriers at the facility level also challenge uptake and continuation of contraceptive use. For example, one study found that youth post-abortion care clients were more likely to choose a contraceptive method if the provider was trained in offering youth-friendly post-abortion care (5). A lack of basic items at the facility such as trained staff, equipment and commodities were also associated with low contraceptive uptake (6) while having health workers benefit from a supervisory visit at health facilities was associated with client uptake of long-acting reversible contraceptives in Togo (7).

There is a growing body of evidence that asserts community engagement approaches that address the drivers of sexual and reproductive health behavior can contribute to increased contraceptive use and a reduction of unintended pregnancies (8). Community engagement can improve reproductive health outcomes by fostering changes with individuals, families and social structures and by linking the community with the healthcare system (9, 10). Family and social changes resulting from community engagement may include increased acceptance for smaller families and birth spacing and contraceptive use (11). Community engagement can also play an important role in improving the quality-of-service delivery and communication between providers and clients (12). Several studies have found that introducing community engagement interventions results in increased knowledge about the importance of healthy timing and spacing of pregnancies as well as increased in contraceptive uptake (13–15). Several evaluations have also shown that community engagement approaches can address social and gender norms including addressing the importance of partner communication and male support for contraceptives resulting in increased contraceptive use (9, 16). Several studies have shown that understanding provider knowledge, attitudes and motivations can inform efforts to strengthen provider performance and improve client-provider communication (17, 18). However, an evidence map of social and behavior change and community engagement interventions for reproductive health found few evaluations of community engagement interventions that addressed health system planning and provider knowledge, attitudes and behaviors towards provision of reproductive health services (19).

West Africa Breakthrough ACTION (WABA) is a regional USAID-funded initiative to increase coordination and effectiveness of social and behavior change (SBC) interventions to drive demand for reproductive health services in four countries: Burkina Faso, Côte d'Ivoire, Niger, and Togo. WABA works in partnership and synergistically with AmplifyPF, USAID's flagship FP project implementing the Integrated Learning Networks (ILN) model to strengthen service delivery in these four countries. The ILN model applies a multisectoral approach that convenes stakeholders at the district level to coordinate resources through ILN technical support committees (a smaller group created from district health committees) to ensure the delivery of high-quality FP services and to build health provider capacity through training on FP counseling, delivery, and quality improvement. Together, these programs aim to improve community perceptions of service quality and FP methods through a community engagement approach. The community engagement approach encompasses three interventions: community dialogues (CDs), health facility site-walk through (SWT) visits, and the development of community action plans. The purpose of the CDs is to create a platform for open discussions and knowledge sharing between the community and the health system. The purpose of the SWT is to enable community members to provide direct observation and feedback to the health workers at the facilities. The purpose of the Community Action Plans is to generate strategies for addressing observed challenges to improve service delivery quality and outcomes. The three approaches work synergistically to address challenges and promote positive service improvements in the health system. Since 2019, when WABA launched these activities, it became clear that additional investments were needed to expand access to the community engagement activities. AmplifyFP began to implement the same series of community engagement activities – meeting with healthcare providers and community members with the objective of building rapport between the clinic staff members and the communities they serve. Members of both projects meet with district-level officials to plan the CD, describe the process of conducting a CD, and select the main theme/topic. During the CDs, groups of up to 100 community members, and clinic staff are each given a chance to share their concerns, desires and frustrations of local FP service access and delivery. An important element of the CDs is male engagement both, in terms of reaching men as heads of households, and as opinions leaders (religious leaders, community chefs, etc.) as they often serve as gatekeepers to FP use. Women's participation in the CDs fosters empathy among community members and supports the identification of needs and solutions that enables the delivery of quality FP services. For example, a pastor donated curtains for the windows in the maternity ward to increase privacy for women in labor. The staff and community groups meet separately to think about their challenges, before sharing them with the other group, in the facilitated dialogue. An intended outcome of facilitation is that communities develop a better understanding of the work of the providers, and the challenges the provider faces in the delivery of quality services, and vice versa, the provider understands the difficulties of community members who come for services including their right to obtain quality FP services.

Following the CDs, a smaller group of approximately 25–30 community leaders, including religious leaders, women's group leaders and local officials, are invited to participate in guided SWT visits at the health facilities where they can ask questions or express concerns on behalf of their constituents related to each health service they visit, engaging in dialogue with facility staff and administration, to learn first-hand about FP services that are offered. The SWT visits are conducted by program staff in collaboration with district and health facility staff. Following the SWT visits, these leaders sit with health facility staff to jointly develop community action plans to prioritize and address the problems identified in the delivery of quality services. The WABA and AmplifyPF theory of change hypothesizes that the joint provider-community action plans will improve the health providers' motivation, attitudes, and self-efficacy to deliver quality FP services. In some instances, the plans also highlight the need for greater involvement of community leaders to challenge and correct rumors and misinformation that some community members have about FP methods or the services themselves. Furthermore, representatives from the facility and community leaders can discuss how to improve services and service use. In 2020, WABA developed a discussion guide to help community groups discuss sensitive topics that may be barriers to increasing use of FP such as gender-based violence, or the lack of couple communication or intergenerational dialogue about family planning between supportive adults and youth. From October 2020 through September 2021, CDs and SWT visits in all 17 ILNs across the four WABA and AmplifyPF program implementation countries were conducted, at a rate of at least two CDs and at least five SWT visits per ILN.

**Figure 1:**
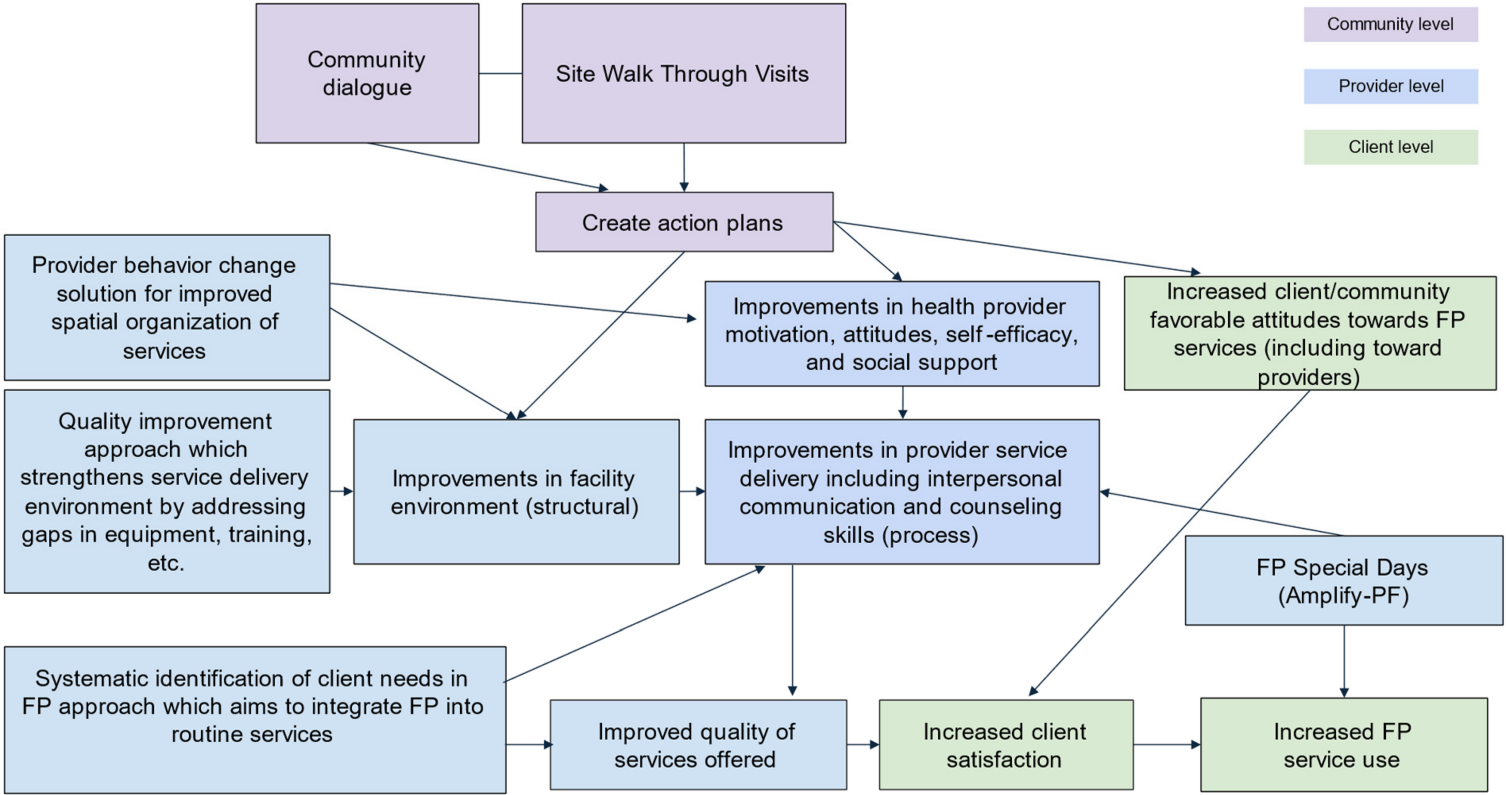
West Africa Breakthrough ACTION and Amplify PF integrated program theory of change.

Guided by the WABA and AmplifyPF program theory of change described in Figure 1, this study qualitatively explores how the community engagement approach was implemented in and around the city of Lomé, Togo, within the context of the broader integrated SBC and service delivery programs. We describe enabling factors and challenges associated with implementation as well as the extent to which the approach achieves the intermediate outcomes of improvements in (a) provider and community perceptions related to FP service delivery (b) the facility environment; (c) provider motivation, self-efficacy, and social support; and (d) improvements in interactions between providers and clients and quality of services offered.

## Methods

### Study design and sample

Using qualitative methods, we conducted a formative evaluation to explore barriers and enabling factors associated with implementation of the community engagement approach, and to understand the perceived effect of the interventions on intermediate outcomes related to community attitudes towards FP services, provider attitudes towards the community, the facility structural environment, interactions between providers and clients and quality of services offered. While the WABA and AmplifyPF interventions focused on four countries, the study took place in and around the city of Lomé, Togo, where the study team identified that the evidence base for SBC for FP service delivery was limited in comparison to the other focal countries. The study team selected intervention study sites and study participants who participated in the interventions and conducted nine focus group discussions (FGDs) with eight to ten community leaders per FGD (Table 1). Study sites were selected if they had participated in all three intervention activities (e.g., CDs, SWT visits, and development of action plans). The purpose of the FGDs was to understand community leaders' experience with the approach and to understand how it influenced their behavior and experiences with the health system. Community leaders interviewed included members of the community health management committees, religious leaders, women's or youth group leaders, mayors, or other government officials such as members of district health teams who had participated in CDs or SWT visits, and/or contributed to development of action plans. The study team also conducted 19 in-depth interviews (IDIs) with a subset of FP service providers who participated in at least one of the interventions (Table 1). Service providers were randomly selected from health facilities participating in the intervention with a FP client volume ranging from two to seven FP clients per day. The purpose of the interviews was to understand the providers' experience with the approach and their reactions to the interventions. We interviewed nurses and midwives with between 10 and 20 years of experience providing FP services primarily at peripheral care units (e.g., social medical centres and primary health units).

**Table 1:** Description of study participants by integrated learning network (ILN) district health facilities.

In-depth interview In ILN district health facilities (*N* = 19)	Focus group discussion Communities (*N* = 9)
Golfe ILN district	
Attikoume (*N* = 3)	Amoutive
Adidogome (*N* = 3)	
Avé ILN district	
Anyron (*N* = 2)	Anyron
Assahoun (*N* = 3)	Aképé
Tovegan (*N* = 2)	Kévé
Agoényivé ILN district	
Togblecopé	Togblécopé
Demakpoè	Sanguerra
Adeticopé	Adéticopé
Lègbassito (*N* = 2)	Lègbassito
CMS Agoènyivé	Danliko

Prior to data collection, the study team developed, piloted, and made subsequent revisions to the interview guides in collaboration with the implementation teams and the Togo-based research partner, the CERA Group. The interview guides explored the following: (1) enabling factors and barriers associated with participation in the approach, which included CDs, SWT visits, and community action plans; and (2) experience with the health system and specifically FP services and any changes that have occurred (community leaders only) and (3) experience with the community and any changes that have occurred (providers only). The interviewers also took part in a weeklong training where they were oriented to the study objectives, research ethics, and qualitative research interviewing skills including role playing to help minimize bias (e.g., avoid asking leading questions).

#### Procedures

Data collection took place from December 2021 to January 2022. AmplifyPF supported the team in obtaining an authorization letter from the Ministry of Health and introduced the interviewers to the health district leadership. Interviewers trained in qualitative methods described the objectives of the research, obtained written informed consent from all participants, and then administered the guide in French to the study participants. Interviews took place in-person. Community leaders were interviewed outdoors in a private, group setting in the community. Health workers were interviewed at the health facility. The FGD and IDIs took approximately 30–45 min to complete. Interviews and FGDs were audio-recorded and transcribed, and research team members took notes on paper during the discussions. Providers were not compensated for their participation. Community members participating in the FGDs were compensated for their transportation to the FGD location. To ensure data quality, the study team reviewed the audio recordings at the end of each day to both identify and address any issues with data quality and to identify when emerging themes reached saturation.

#### Analysis

The study team developed codes based on the theory of change and study questions. Additional codes were developed by applying open coding on a subset of transcripts to co-construct new themes. The study team applied codes using Dedoose, an online, collaborative qualitative research coding and analysis platform (20). Prior to applying the codes across all transcripts, a subset of transcripts was coded by the team to ensure agreement in coding. Inconsistencies in how codes were applied were resolved after discussion with the broader study team. The team then used thematic analysis to compare findings for each intervention by study participant (e.g., community member/provider) to highlight common and divergent results across themes. All coding and analysis were conducted in French, with data excerpts translated to English for publication purposes.

#### Ethical approval

The Ministry of Hygiene and Public Health and Universal Access to Care in Togo provided approval for the study and consent forms (No. 033/2021/CBRS). The study also received approval from the Population Council Institutional Review Board in the United States (No. 985).

## Results

We first present enabling factors and challenges associated with CDs, SWT visits, and the development of community action plans and then describe the perceived effect of these interventions on intermediate outcomes related to community attitudes towards FP services, provider attitudes towards community members, the facility structure environment, and interactions between providers and clients.

### Enabling factors and challenges associated with implementation of the community engagement approach

#### Community dialogues

Study participants described participation in the CDs as including a range of stakeholders, including representatives from the municipality, chieftaincy, religious leaders, women, and youth leaders, as well as nurses and midwives from the health centers and members of the WABA and AmplifyPF teams. Community members interested in the CDs joined as well, as the sessions were public and held outdoors. Most of the CDs occurred over approximately two hours and took place at the health facility or in the homes of local chiefs. While few specific roles were assigned during the CDs, participants noted that CD participants were expected to relay information to their respective communities while providers were expected to increase awareness about FP methods and services as well as broader factors that affect FP use through the CD activities. Topics discussed during the CDs included addressing rumors and the communities' perceptions of contraception particularly among men who often serve as gatekeepers to FP use and discussing the advantages of different contraceptive methods. CDs also discussed how clients were received at the facility and an inventory of the general challenges faced by the health facility, such as a lack of basic infrastructure (e.g., sink, light bulbs, benches, curtains for privacy) and reasons for low attendance. From October 2020 through September 2021, approximately 347 men and 424 women participated in CDs in Togo.

#### Enabling factors associated with community dialogue implementation

Study participants identified several factors that facilitated implementation. First, health workers acknowledged the CDs provided an opportunity for them to engage with men on FP topics, which is not always possible at the health facility when women attend alone. Second, the CDs fostered a sense of partnership between the communities and the providers by creating a joint forum for facilitated discussion, and by allowing select community members to act as liaisons between providers and the broader community. However, participants description of this partnership reflects a skewed interest centered around helping providers reach the community more so than empowering the community to mobilize for improved health access.

« after our CD, the midwives felt that…they now have a representative at the community level and at every activity if they go…If they want to promote an activity, they always use us. So…we can say that we have a partnership relationship with the agents, the providers…at the medical center level » (male community leader, Sanguéra)

#### Challenges associated with community dialogue implementation

Study participants identified several challenges related to implementation of the CDs. As some of the community activities took place in the first two years of the COVID-19 pandemic, several participants noted that this made it difficult to organize community events, as the government encouraged physical (social) distance and discouraged in-person gatherings. There were mixed perspectives on the duration of the CDs. Several providers noted delays in starting the CDs and reported that their overall duration was too long, while community members reported that the time allotted did not always allow for in-depth discussion about FP topics. Finally, several community members acknowledged limitations in participation; Catholic religious leaders chose not to participate in the meetings given the activities' focus on promoting the benefits of voluntary modern contraceptive use, not just natural family planning methods.

“…the priests there and the pastors there … They gave a verse [from the Bible]: Go and procreate like the sand of the sea, which God said, that was their reasoning» (male community leader, Keve)

#### Site walk throughs

Following the CDs, the local administrative authorities in collaboration with the heads of the health facilities (FP focal point and health facility manager) proposed SWT visit participants which included both people who participated in the CDs (e.g., traditional chieftaincy, religious leader representatives, community health workers, and health staff) as well as some local executives and business leaders who may not have attended the CDs. From October 2020 through September 2021, approximately 251 men and 210 women participated in SWT visits in Togo. Study participants described the SWT visits as an opportunity for community representatives to visit the health facility and gain a better sense of the challenges faced by health workers as well as to share their perspectives with health workers regarding the quality of FP services offered. The SWT visits occurred, on average, only once per facility, in all facilities offering FP and maternal health services, usually within three months following a CD, for a duration between one and four hours.

#### Enabling factors associated with implementation of site walk through visits

The main factor that encouraged the SWT visits was that the CDs informed community representatives about the SWT visits and encouraged them to come and see the difficulties encountered at the various health centers first-hand. For many participants of the CDs, the SWT visits was the first opportunity to enter the health center and ask questions of the FP service providers. This also enabled health workers to understand FP information needs from the community perspective and helped to reinforce the challenges prioritized in the CDs.

#### Challenges associated with implementation site walk through visits

Overall, there were few challenges reported with the SWT visits. Some community members interviewed were not familiar with the activity as only a subset of those present for the CDs participated in the SWT visits. While youth leaders participated in some SWT visits, one community member noted that youth did not participate in the SWT visits because they were not considered responsible or capable of contributing financially to facility improvements. Several community members also reported that incentives should be provided such as a transportation stipend and refreshments to encourage participation in the SWT visits**.**

#### Community action plans

In many instances following the SWT visits, a self-identified monitoring committee developed a community action plan that addressed deficiencies found in the facility in a prioritized list of action items. At the time of data collection, action plans were at various phases of implementation for each facility and included needs for medical equipment, and structural support (e.g., birthing tables, screens for windows, etc.) as well as community outreach sessions on the benefits of using FP. Regarding client-provider interaction, activities focused on increasing supervision and feedback related to how providers welcomed clients at the facility. In Togo, out of the 37 action plans developed, 24 were implemented while 13 had not yet been implemented at the time of data collection.

#### Enabling factors associated with implementation of the community action plans

Community members and health workers acknowledged that the community action plans provided a platform to mobilize community members to address the challenges faced at the health facilities including contributing to a pooled funding mechanism that could be used to address structural challenges at the facility. In instances where district supervisors and WABA and AmplifyPF trainers organized follow-up after meetings to discuss progress, community members acknowledged how the follow-up facilitated execution of the activities outlined in the action plans. The action plans also served as an accountability mechanism that enabled periodic monitoring of implementation.

#### Challenges associated with implementation of the community action plans

Study participants reflected on several challenges that emerged with the execution of community action plans. Several community members mentioned that in some cases, committees were not formed or did not meet following the SWT visits to manage the identified activities.

«Yes, for example, here when we elaborate the action plans, the follow-up is not as good. For example, the first time we did the site visits, we had elaborated the action plans but there was no follow-up. In the second round, action plans were developed again, but so far there has been no follow-up. The follow-up is not that» (female health worker, Adéticopé)

Both community members and health workers reflected on the challenges in mobilizing resources to implement activities outlined in the action plans. Several participants noted that a lack of coordination within the community-based monitoring committee contributed to insufficient financial resources to carry out the actions discussed.

« Yes, a lot of activities, first the contributions, we did not start … After the departure of those who came to make the dialogue, after there was no more follow-up or disturbances, everyone remained cloistered in his corner» (male community leader, Keve)

### Perceived effect of the community engagement approach

#### Community has favorable attitudes towards FP services and providers

Health workers and community leaders reflected on the perceived positive effects of the interventions, including how the CDs helped to dispel FP-related rumors in the community and helped to address resistance to FP among some men in the community.

“During this activity, what was important to me was that we dispelled the rumor about the IUD (Intrauterine Device), because many women had reservations about it because they said that when you put the IUD in your belly, it goes into your lungs. So they were really afraid to practice this method. …With this presentation…the women who were on IUDs who were in the group had testimonies that made others say that therefore it was a good method, [and] so it was rumors. That’s what really amazed me.” (female health worker, Togblecope)

The CDs also raised awareness among community members about the unique needs of youth who use FP.

“….and then we had a dialogue between the youth. That is to say… the young people do not share the same opinion with the old people. They have secrets that they can deliver between them young people, as there is … the distrust or what we call it uh, the generational conflict. That’s why there is no waiting ground. Now the day we were taught how to live in collaboration with the elderly, these are the points that marked us “ (male community leader, Keve)

Increased community awareness of the challenges faced in the health facilities observed through the SWT visits also helped to generate a more positive view of providers and the challenges they face in delivering services.

“ The subjects I was talking about are the activities that we identified at the level of community dialogue, which we have taken up here, which we have taken up and deepened. Because here, at the guided tour, the community had come to visit the center and they saw in what condition we work. They saw that the material was not sufficient - the insufficiency of material that we noted - and the working conditions too, and it was necessary to review certain working conditions. So with that we made a plan of action - first the sensitization of the community now on the plan of insufficiency of material ….. We listed the materials that are, that are not in good condition or that do not even exist, and the community has taken everything and has even promised to make a mini project or a budget that they will present to different donors to see to what extent they can have this material and put it at our disposal” (female health worker, Adidogome Satchivi)

Health workers also reported that SWT visits provided an opportunity to explain challenges in providing services, such as the absence of equipment, and how these challenges contributed to patient dissatisfaction.

« …I will say what I appreciated. The visit made it possible to see the reality of the center and thus also to make the community participate in the needs of the center. Because when we often go there, we usually accuse the people who are working there, but these people are struggling with the means they have, and we say every time that it is the state that has to provide when the providers are making a small gesture to improve the daily life of our patients who come there. Also, at the [health facility] level we saw the absence of a waiting room for our, our pregnant women who arrived, therefore the need there also was enumerated and I was so moved that at a big center we do not find especially the oxygen bottles, that was something that touched me a lot and I am sure that after an awakening each one will put of his hand out so that the center can have a good face. » (male community leader, Togblékopé)

#### Provider motivation, attitudes towards community members, self-efficacy, and social support

Providers reflected on how the CDs helped them to better understand the needs of the community, both in terms of the way they welcome clients which was routinely expressed during the CDS as well as understanding their specific needs and concerns which served as a source of motivation in their work.

*« Well… I would say that… during this activity the other day, the exchanges gave us… uh… the opportunity to uh… listen more to the, the, the… the points or the… needs; not the needs, the worries, the concerns of the population regarding family planning. Because…there are people among them who talked about their own experience with family planning, so it allowed me to…to [Inaudible]…it allowed me to enrich more…my knowledge about the method, the advantages and disadvantages of family planning. »* (female health worker, Adidogomé)

However, providers did not directly describe how activities increased their sense of self-efficacy to perform their duties nor social support that was attributed to the interventions.

#### Improvements in the facility structure environment

Providers reported that some of the activities resulted in a continuous supply of equipment and materials to health facilities generated through community contributions identified in the action plans. Community members acknowledged the approaches facilitated outreach sessions that increased awareness on the benefits of FP, but some felt there were more challenges in addressing needs that required additional resource mobilization as only about half of those that included the need for structural improvements had addressed the need.

“What is good in the guided site visits is that it is the population itself that detects the problems related to the health facility and the population or the participants give approaches of solutions and plans to be put in place to get there. So, in order not to talk too much, I would say that in the health center, following this guided site visit, our structures are well equipped with screens and mosquito nets, and this is a gift from the community,” (male health worker, Anyron)

#### Improvements in interactions between providers and clients and quality of services offered

Community members reported that dialogues led providers to offer clients an improved welcome at the health facilities. Improvements implemented at the facility provided community leaders with a sense that their feedback was heard and addressed, which in turn created trust between the community and the health workers. In addition, community leaders perceived that the CDs led to a decrease in community fears/constraints toward FP providers and services.

« with the arrival of = WABA=, it opened our eyes, and now women are no longer afraid of midwives, even for small health problems they go to the midwives to check if it is a pregnancy and the nurses reassure them that it is not a pregnancy or it is a pregnancy; they do not try to take products without advice, so they do not do what they used to do before when they avoided going to the health center. » (male community leader, Togblekope)

Providers noted that the activities enabled them to better understand how to address misunderstandings related to FP so that they could provide better quality of care through improved counseling with clients. For example, some clients believed that they would no longer be able to have children if they adopted a method such as injectables or implants and providers were able to explain through more in-depth counseling approaches how the FP methods could be reversed.

“On a general level, she (the client) said that there were many people who said that it is like that when you (use FP) after you are not going to give birth anymore… We told them that this is not the case… So they understood too. For the Depo-Provera they also asked questions that they learned that this is how some people do it and then when they want to get pregnant, they stop but the pregnancy does not come. We said yes, that’s true, but it’s because the product is in the blood in some of them, the product has to finish completely in the blood before the pregnancy comes, but in some of them, it’s only appointment they miss, they get pregnant. So each one has… (silence) each organism with its own temperament, so they have understood too.” (female, health worker Togblecope)

## Discussion

This study qualitatively explores how a community engagement approach influences provider and community perceptions related to FP service delivery in and around the city of Lomé, Togo, within the context of a broader integrated SBC and service delivery program. Our findings, organized by the project theory of change, contribute to the growing body of literature exploring how community engagement can be used to collaboratively work with communities and health providers to identify problems and implement solutions to improve FP service quality. We identified enabling factors and challenges associated with implementation, as well as perceived effects of the approach on program outcomes.

## Enabling factors and challenges associated with implementation

Our study sheds light on how CDs, SWT visits, and the development and implementation of community action plans synergistically support collaborative action between communities and health workers to increase mutual understanding of their respective needs related to FP programs and services. Consistent with previous research, we found that CDs served as an effective strategy to build partnerships between communities and the health centers enabling providers to address gender norms by reaching male audiences who are not always reached through the formal health system with information related to reproductive health services and shifting norms whereby men were previously seen as gatekeepers to obtaining FP methods but now play a more supportive and enabling role (21). We also found that interactions between community members and health workers through SWT visits fostered greater community empathy for workplace challenges, thereby contributing to increased trust between the community and providers as shown in previous evaluations (22, 23). Community members were also able to express their FP concerns (e.g., rumors and misinformation as well as valid concerns related to specific contraceptive methods) with health providers through the community engagement activities (24). As observed in previous research, efforts to make structural improvement to the health facilities while, reinforced by the ILN technical committees, varied based on community leadership and were stymied by the need to mobilize additional support including financial resources (25). Previous research also suggests strengthening district health management teams to supervise health workers and follow-up on community concerns may help to generate a sustained response from community structures (26).

## Perceived effects of the community engagement approach on FP services

Evidence generated through the FGDs with community members found that CDs helped to create a greater understanding of youth contraceptive needs, and the need to provide greater support and information to youth related to sexual and reproductive health (SRH), particularly at the community level. WABA activities aimed to foster improved support for youth SRH through engagement at the community level, which is an approach that may support community members to raise health concerns with providers (27). Programs should consider developing specific interventions at the community level designed to address youth needs such as separate youth centered meetings or youth-only SWT visits to address challenges faced by this population and generate sustained support (28–30). Breakthrough ACTION through its mass media campaign Merci Mon Héros (MMH, Thank You My Hero) which targets youth and adults, provides another example of how programs can promote an environment conducive to young people's access to FP/RH services in the four WABA and AmplifyPF countries.

Consistent with previous studies, our findings suggest that the approach also helped to improve client-provider interactions for FP services including improved quality of care (31, 32). However, additional research is needed to understand if the WABA approach has contributed to increases in contraceptive uptake. Previous studies have shown mixed results regarding the implementation of similar approaches and improved health outcomes (33–36)**.** Additionally, given the limited adherence to community action plan activities that address structural challenges at the facility level, there may be limits in the extent to which this approach can be used to address broader health system improvements outside provider behavior and community engagement, particularly in low-resource settings (37, 38). Finally, this study finds evidence that the implemented program fostered a sense of accountability from the community towards the facility and providers, yet evidence of increased accountability from the providers to the community is more limited. Programs may also want to consider layering a CSC approach into the existing program to strengthen collective action and ensure that communities make continued progress towards achieving their planned activities (34).

## Limitations

Our study assesses the perceived effect of approaches aimed at facilitating interactions between community members and health workers to improve health provider behavior and FP service quality. Our study did not interview supervisors of health workers nor policymakers to assess their role in influencing health workers performance, which may limit our understanding of the role that contextual factors play on study outcomes (39, 40). In addition, while the current study provides a qualitative exploration of a community engagement approach and its perceived effectiveness on FP programs and services, findings related to providers motivation and attitudes were limited. Incorporating quantitative measures that serve to evaluate provider motivation, attitudes towards community members, self-efficacy, and social support would have strengthened the study (41, 42). More evidence is needed from the francophone West Africa region to quantitatively assess the effect of these approaches including the development and utilization of validated measures that establish linkages between community participation, empowerment, and quality of care (43–47).

## Conclusions

Findings from this study aim to inform ongoing implementation and to build the evidence base for community engagement approaches in West Africa, where evidence is limited. Our study identified enabling factors and challenges associated with program implementation of three integrated interventions CDs, SWT visits, and the development and implementation of community action plans. We found CDs and SWT visits were promising platforms for addressing community needs for information about FP services, building empathy for providers and fostering trust in the health system. Completion of activities identified through the community action plans had mixed results. While planned activities addressing information needs were largely completed, there was less evidence demonstrating community action plans generated financial resources necessary to address structural challenges at the facility level. Future programs should consider incorporating additional mechanisms for monitoring of community action plans and providing support to address structural challenges at the facility level particularly those that require financial resources through mechanisms such as village savings and loans associations.

## Data Availability

The raw data supporting the conclusions of this article will be made available by the authors, without undue reservation.
